# N-doped graphene-based copper nanocomposite with ultralow electrical resistivity and high thermal conductivity

**DOI:** 10.1038/s41598-018-27667-9

**Published:** 2018-06-18

**Authors:** Liang Zheng, Hui Zheng, Dexuan Huo, Feimei Wu, Lihuan Shao, Peng Zheng, Yuan Jiang, Xiaolong Zheng, Xinping Qiu, Yan Liu, Yang Zhang

**Affiliations:** 1 Laboratory for Nanoelectronics and NanoDevices, School of Electronic Information, Hangzhou Dianzi University, Hangzhou, 310018 China; 2 Institute of Materials Physics, Hangzhou Dianzi University, Hangzhou, 310018 China; 3 Department of Chemistry, Tsinghua University, Beijing, 10084 China; 4 Chemistry and Biochemistry Department, California State Polytechnic University-, Pomona, CA 91768 USA

## Abstract

Nanocomposite with a room-temperature ultra-low resistivity far below that of conventional metals like copper is considered as the next generation conductor. However, many technical and scientific problems are encountered in the fabrication of such nanocomposite materials at present. Here, we report the rapid and efficient fabrication and characterization of a novel nitrogen-doped graphene-copper nanocomposite. Silk fibroin was used as a precursor and placed on a copper substrate, followed by the microwave plasma treatment. This resulted nitrogen-doped graphene-copper composite possesses an electrical resistivity of 0.16 µΩ·cm at room temperature, far lower than that of copper. In addition, the composite has superior thermal conductivity (538 W/m·K at 25 °C) which is 138% of copper. The combination of excellent thermal conductivity and ultra-low electrical resistivity opens up potentials in next-generation conductors.

## Introduction

Copper is the most common conductor used in electrical energy distribution, data transmission field, and semiconductor industry due to its excellent heat and electrical conductivity. Modern industry is witnessing an increasing demand for better heat and electrical conductive materials at the level beyond copper. Nanostructured carbon materials, such as carbon nanotubes (CNT) and graphene, are emerging as new conductive alternatives due to their excellent electrical, thermal and mechanical properties^1,2^. Furthermore, combing copper with high performance nanostructure carbon materials could, theoretically, create a novel composite conductor with a room temperature resistivity far below that of conventional metal copper (Cu)^3^. However, achieving such a room temperature conductivity which is estimated by the theoretical model to be 50% below that of Cu remains great challenges. These challenges come from ballistic conducting CNT preparation and interface controlling between CNT and Cu matrix^4,5^. One promising alternative way to fabricate this kind of ultra-low resistive composite conductor is to deposit graphene on copper matrix^6,7^.

Since the discovery of unique heat conduction properties of graphene by the UC Riverside group^8^, the thermal conductivity of the composite with graphene has attracted great attention. The pioneer work by Balandin stimulated research on development of these composites with graphene enhanced thermal and electrical properties, which may have many practical applications^9–11^. Although suspended graphene has very high in-plane thermal conductivity (~5000 W/m.K)^8^, graphene placement on other substrates results in the degradation of the composite thermal conductivity (~600 W/m·K on SiO_2_^12^ and ~460 W/m·K on copper^13^, respectively), which raises great concerns for its applications in nano-electronic and nano-optoelectronic devices^14^. The theoretical calculations indicate that the thermal conductivity of the graphene-based metal composite is dependent on the properties of interfacial between graphene and metal^15,16^.

Many efforts have been made to develop the graphene/graphite platelet-copper composites with improved thermal properties^17,18^. Nitrogen-doped graphene sheets (NGS) composited with Cu matrix have shown thermal conductivity of ~500 W/m·K^19^, which is higher than that of pure graphene composite with Cu matrix. However, electrical resistivity of these composites is still higher than that of copper. Reports showed that both nitrogen-doping contents and types of nitrogen bonding in NGS played an important role on electric and thermal properties. For example, quaternary N structures that resulted from the replacement of C atoms in hexagonal rings by nitrogen atoms led to enhanced high performance electric conductivity and thermal conductivity^20,21^.

**Figure 1:**
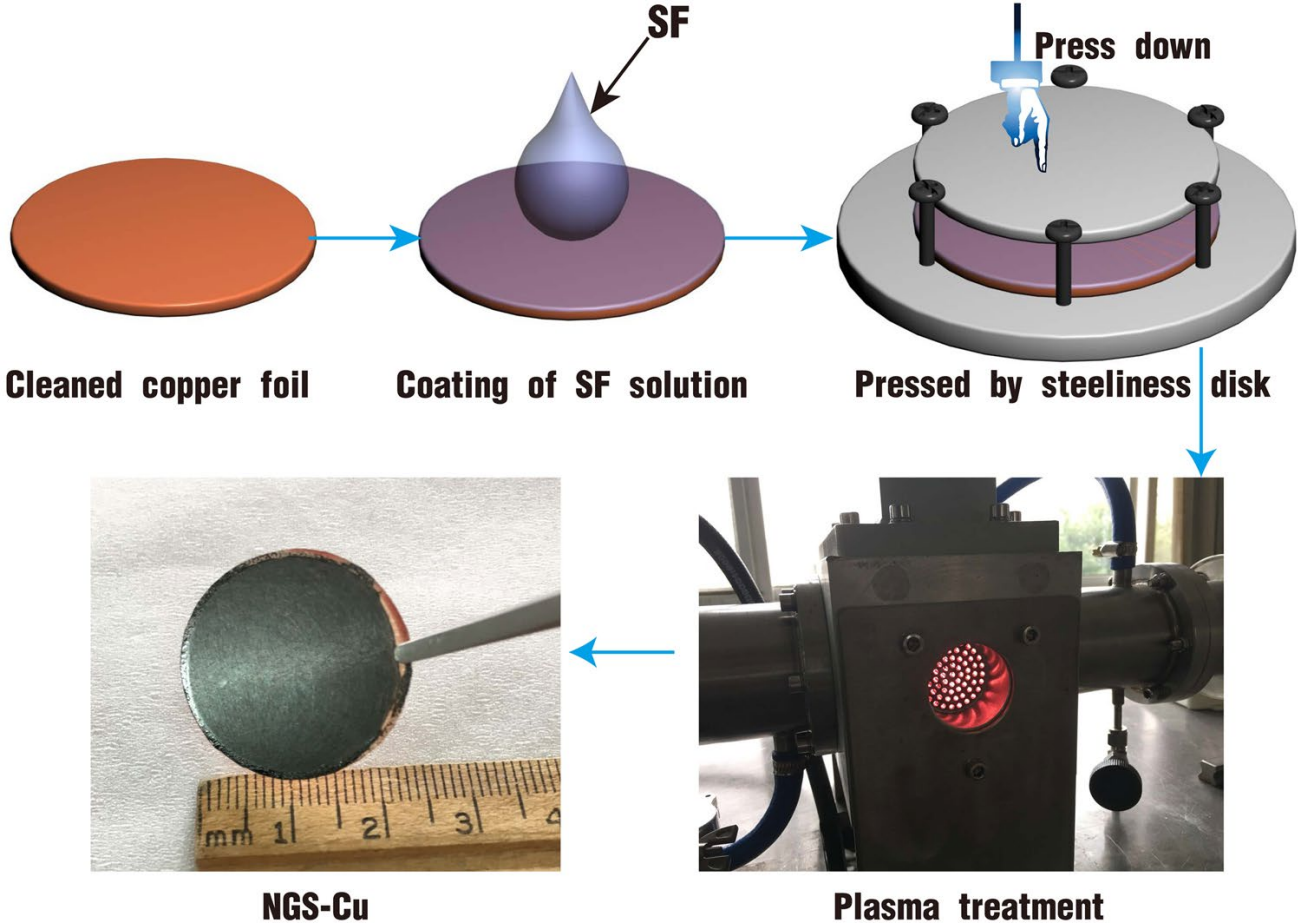
Illustration of NGS-Cu composite fabrication. Plasma treatment was performed with a home-made reactor with a diameter of 45 mm quartz chamber at 2.45 GHz with microwave generator capable of generating 1.5 kW power which determines the maximum size of the Cu foil that can be used.

Here, we report a simple route to fabricate NGS-Cu nanocomposite conductor with ultra-low electrical resistivity and high thermal conductivity using silk fibroin (SF) as precursor. Briefly, Silk fibroin was used as the carbon precursor and its solution was spin-coated on a clean copper foil. Following the mounting and microwave plasma heating (MPH) treatment of SF/Cu, NGS-Cu nanocomposites were produced (illustrated as Fig. 1; the detailed description of NGS-Cu fabrication is included in the Methods: Preparation of Silk Fibroin Solution and Fabrication of NGS-Cu Composites). Overall, the synthesis of graphene, doping of nitrogen, and formation of nanocomposites were completed in a single step process. What is more important is that at room temperature, the synthesized NGS-Cu composite owns a resistivity of 0.16 μΩ·cm which is only 7.6% of pure Cu; whereas the thermal conductivity of the NGS-Cu composite is 538 W/m·K which is 138% of pure Cu.

## Results

### Electrical Conductivity of NGS-Cu composite

**Figure 2:**
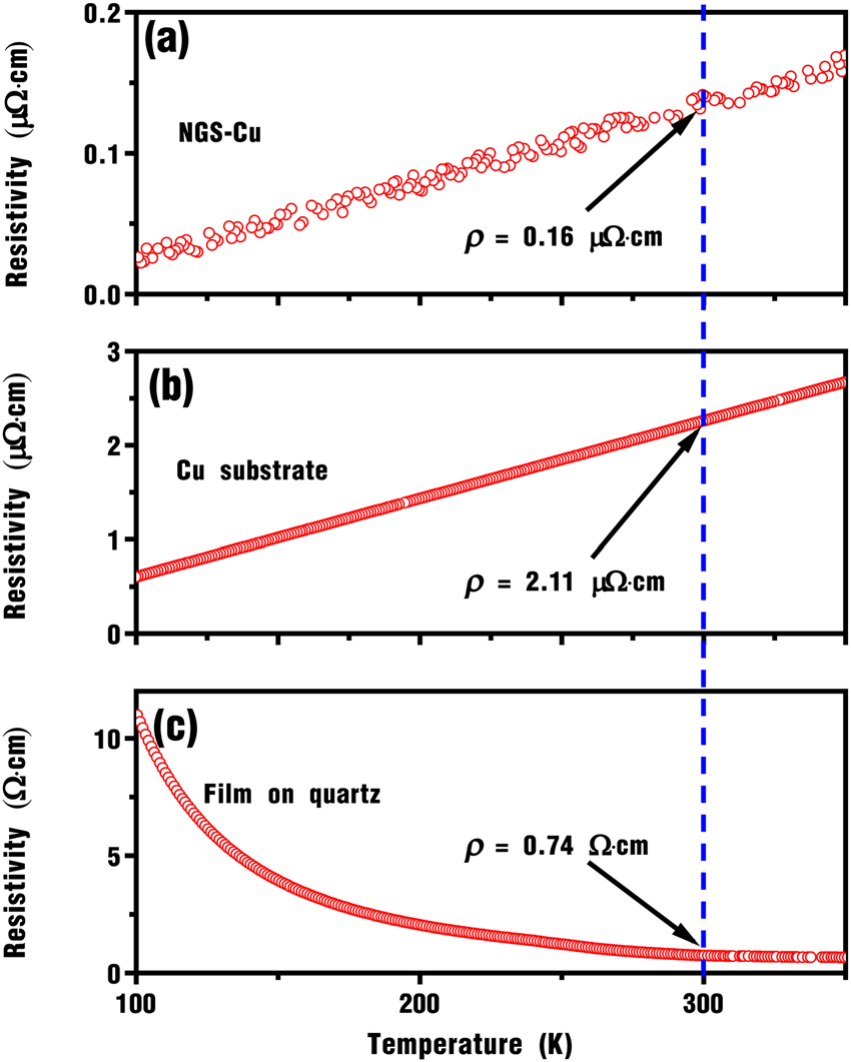
The temperature-dependent electrical resistivity of samples. (**a**) NGS-Cu composite. (**b**) Cu substrate. (**c**) NGS film on quartz.

The standard dc four-probe method was employed to investigate the temperature-dependent electrical resistivity of the NGS on Cu, pure Cu substrate and NGS on quartz in the temperature range of 100 to 350 K. Evidently, the resistivity of the NGS on Cu exhibits metallic behavior similar to Cu and it increases with the temperature increase. At 300 K, the resistivity of the NGS-Cu composite is 0.16 μΩ·cm (Fig. 2(a)), which is only 7.6% of the value of pure Cu substrate 2.11 μΩ·cm (Fig. 2(b)). Oppositely, the resistivity of the N-graphene sheets fabricated on quartz substrate shows a typical semiconducting behavior (Fig. 2(c)). The resistivity value at 300 K is 0.74 Ω·cm, which is much larger than that of the composite and pure Cu in the whole temperature range. These results strongly suggest that the NGS-Cu composite is an ultra-low resistivity conductor.

### Thermal conductivity of NGS-Cu composite

**Figure 3:**
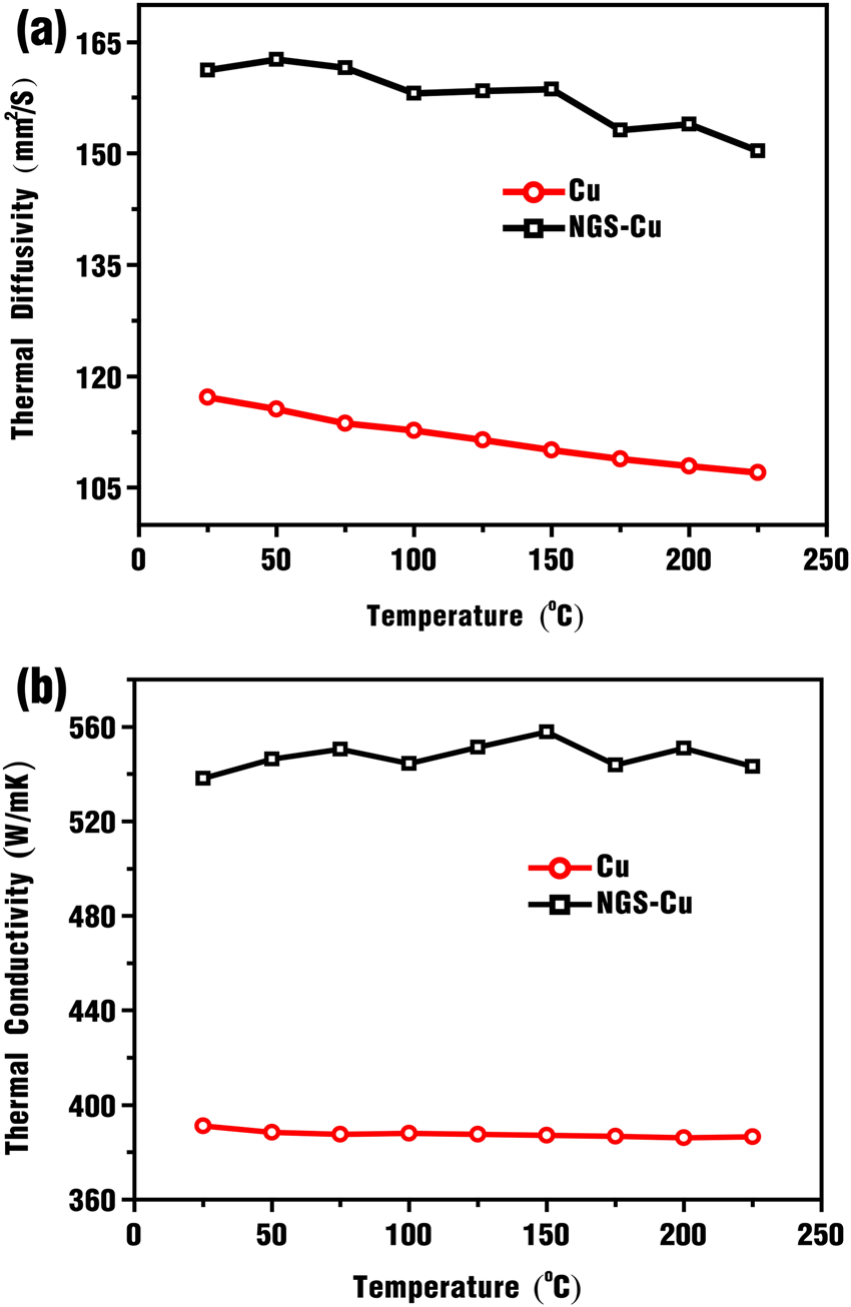
Thermal properties of NGS-Cu composite and reference Cu. (**a**) Thermal diffusivity. (**b**) Thermal conductivity. The measured error is ±3%. The thermal conductivity was determined from the equation *K* = *αρC*_p_, where *ρ* is measured to 8.9 g/cm^3^ by Archimedes method and *C*_p_ is measured by differential scanning calorimeter, the details are summarized in Methods.

The measurements of the thermal diffusivity were carried out using the “laser flash” method which gives the cross-plane thermal diffusivity, ***α***, of the sample. The thermal conductivity (***K***) was determined from the equation of ***K* = *ραC***_p_, where ***ρ*** is the mass density of the sample and ***C***_p_ is the specific heat of the sample measured, respectively. The details of the measurements are summarized in Methods. Figure 3 presents the temperature-dependent of thermal diffusivity and thermal conductivity of Cu and NGS-Cu composite, indicating that a large improvement in thermal diffusivity and conductivity of NGS-Cu composite over Cu foil. For example, the thermal diffusivity of NGS-Cu jumps from 117 mm^2^/S of Cu to 161 mm^2^/S at 25 °C, while the thermal conductivity of NGS-Cu composite is 538 W/m·K at room temperature which is 138% of Cu. The behavior of the temperature-dependent thermal diffusivity and conductivity of NGS-Cu composite is similar to that of reference Cu in the temperature range of 25–225 °C.

### Characterization of NGS-Cu composite

**Figure 4:**
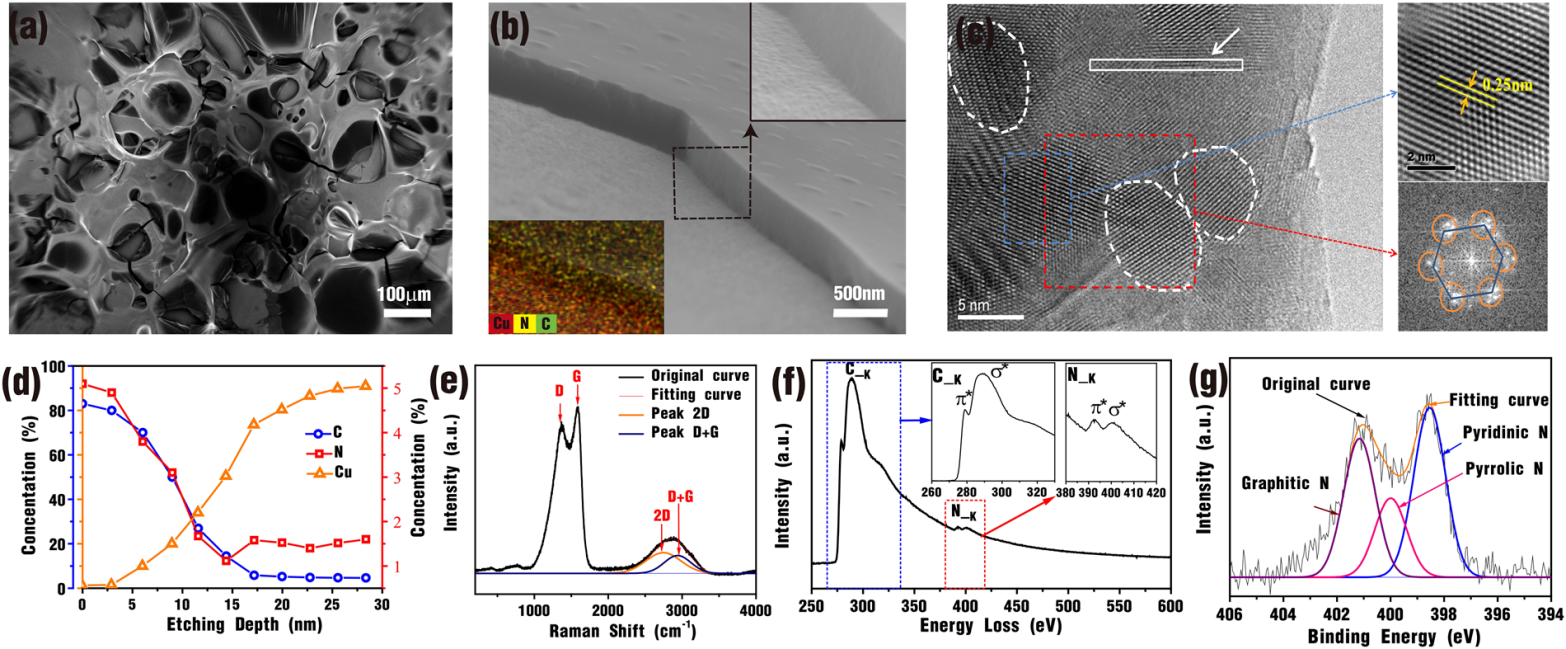
Structural characterization of NGS-Cu sample. (**a**) SEM image of the top view the NGS-Cu composite sample. (**b**) SEM image of the sectional plane of the NGS-Cu composite sample. The inset in the right corner shows the enlarged view of the place circled by black dotted line. The inset in the left corner displays the mapping including Cu, C and N. (**c**) HRTEM image of sample and corresponding fast Fourier transform pattern. (**d**) XPS depth profiles of NGS-Cu composite. (**e**) Raman spectrum of the NGS-Cu composite. (**f**) EEL spectra of the NGS-Cu composite. The C-K and N-K edge are enlarged in the inset. (**g**) High-resolution N1s XPS spectra of NGS-Cu composite.

The fabricated NGS-Cu composites were analyzed and characterized by several spectroscopic techniques including field emission scanning electron microscope (FE SEM), high resolution transmission electron microscopy (HRTEM), electron energy loss spectrum (EELS), energy dispersive spectrometer (EDS), Raman spectroscopy, X-ray photoelectron spectroscopy (XPS). Figure 4(a) shows a FESEM image of the NGS-Cu composite which clearly indicates the N-graphene film on top of Cu foil is a layered structure with crumpled flaky wrinkles. The area of well-shaped sheet is larger than 100 µm square (Fig. S3). In addition, the film with a thickness of ~500 nm is homogeneously coated on the surface of Cu illustrated by the cross sectional image (Fig. 4(b)). The enlarged view of the dotted square area displays a perfect contact between N-graphene film and Cu substrate. The EDS mapping of the interface is also inserted in the bottom-left corner of Fig. 4(b), confirming the presence of Cu, C and N in this area, and the diffusion of Cu into the N-graphene sheets from the Cu substrate. The copper atom diffusion is also confirmed by XPS depth profiling measurements, shown in Fig. 4(d). It is observed that the content of Cu increased with the increases of etching depth, while a decreasing trend is observed for C and N atoms. Therefore, it is speculated that an interface with tens of nanometers contains a significant amount of Cu, C and N atoms.

Raman spectroscopy is the most efficient way to provide a rapid and reliable structural characterization of carbon-based nanomaterials. Figure 4(e) gives the representative Raman spectrum of the NGS-Cu composite which displays two prominent peaks (of the D band and G band) centered at 1365 and 1590 cm^−1^ as well as a weak and broaden band centered at 2848 cm^−1^ (originated from the overlapping of ~2735 cm^−1^ (2D) and ~2945 cm^−1^ (D + G) two bands). Furthermore, two peaks which appear in the lower wavenumber (<1000 cm^−1^) correspond to the static vibration mode of the doped nitrogen. All of these spectral features are the characteristics of high content CN_x_ within graphene structure^22,23^. The G band corresponds to the first-order scattering of E_2g_ mode of sp^2^ carbon domains, whereas the D band is assigned to the structural defects/disordered of sp^2^ domains. The intensity ratio of the D band and G band (*I*_D_/*I*_G_) is 0.85, indicating the degree of disorder within the graphitic carbon^24^. The 2D feature corresponds to the overtone of the D band; its shape is strongly dependent on the number of graphene layers in a given sample. Therefore, the broader and relatively low intensity of 2D peaks indicates the fabricated NGS-Cu composite contains multi-layers of graphene sheet, which benefits thermal transport on large substrate surface area^17^.

Further, the HRTEM image (Fig. 4(c)) displays the NGS synthesized has well-defined crystalline structures with the interlayer lattice spacing between (100) planes of 0.25 nm and six-fold symmetry from the fast Fourier transform (FFT) computation. In addition, Fig. 4(f) gives the EELS result of the rectangle place marked by the white arrow in the HRTEM image. From the peak area of N-K edge and C-K edge, the N content was quantified to be around 6.0 _atom_.% which is consistent with EDS result (Fig. S4). As seen in the C-K edge, the ~278 eV peak is due to the electron transition from 1 s to π*, while the broad peak centered at 289 eV corresponds to the σ* region. It can be deduced that the synthesized N-graphene is made of graphitic network with sp^2^-type bonding. The fine structure of the N-K edge is enlarged in the inset of Fig. 4(f), displaying a triangular σ* band at ~400 eV, which is characteristic of CN_x_ materials. Another structural feature observed is at 393 eV, which could be attributed to the π* states of the pyridine, pyrrolic or graphitic-like configuration^25^.

XPS measurements were performed to determine the composition and chemical bonding of the N-graphene. A predominant graphite C1s peak at ca. 285 eV and N 1 s peak at ca. 400 eV are observed in the Fig. S5 with the nitrogen content to be further calculated as 5.2 _atom_%. The N 1 s high resolution XPS spectrum (Fig. 4(g)) can be deconvoluted into signals for pyridinic N centered at 398.5 eV, pyrrolic N centered at 399.9 eV and graphitic N centered at 401.2 eV, respectively. High percent of graphitic N (∼35%) in the doped graphene sheets film contributes the benefits of electrical conductivity and thermal conductivity of composite^20,21,26^.

## Discussion

In this work, the synthesis of graphene layers, nitrogen doping and performing nanocomposite with copper were simultaneously done in one step process, which benefited from the unique structure and properties of silk fibroin protein and our microwave plasma heating process. Not only is the silk fibroin protein carbon precursor, but also is nitrogen source for doping. The MPH feeds the reaction conditions for the formation of the NGS-Cu nanocomposite. However, the formation mechanism of the nanocomposite is unclear at present.

The as-synthesized NGS-Cu nanocomposite shows excellent thermal conductivity and ultra-low electrical resistivity. Although the mechanism of electrical or thermal conductivity enhancement is not completely understood yet, we believe that the interfacial structure and interaction of NGS-Cu in the nanocomposite are key factors of controlling interfacial thermal transfer and electrical conduction. Most of studies on the interfacial interaction of graphene-Cu indicate that a weak interaction, e.g., *Van der Waals* interaction, termed Physisorption^15,27,28^, is present between Cu and NGS. In such a case, the phonon scattering mechanism is responsible for the interfacial thermal transfer. If the weak interaction exists in the graphene-Cu interface, both of thermal resistance and contact resistance are expected to be large^29,30^. Obviously, this kind of weak interaction mechanism cannot account well for our results that NGS-Cu nanocomposite processes ultra-high electrical and thermal conductivities. It is, therefore, expected that there exists a strong interfacial adhesion interaction^31,32^, e.g., chemical bonding, between N-doped graphene and Cu matrix. This kind of strong interaction may be originated from nitrogen doping effect or even combined effects of dopant atom-coordinated bonding and mechanical interlocking^19,33^. In such a matter of strong interfacial adhesion interaction, electrons possibly are the main heat carrier in NGS-Cu interfacial transfer^19,30^. Doping graphene with nitrogen makes the *Fermi* level of graphene shift upwards, enlarging the difference between graphene and Cu work functions. A strong electrostatic interaction induced by the charge transfer process is expected to be present between the graphene and Cu substrate^15,34^, and the resultant electronic coupling at the interface could help to elevate heat dissipation efficiency^15,30,35^. In addition, the surface orientation of Cu crystallites might affect the interfacial interaction^34^. Based on the literature study, we deduced that the well bonded NGS-Cu interface is expected to be beneficial for thermal and electrical conductance enhancement in the NGS-Cu nanocomposite.

In conclusion, a new NGS-Cu nanocomposite material with high thermal conductivity and low electrical resistivity was successfully fabricated using silk fibroin (SF) as precursor with microwave plasma treatment. It is expected that the NGS-Cu nanocomposite holds great promises for its applications in advanced high performance electronic and optoelectronic devices, and opens a door for intensive and in-depth researches of new conductors with both high electrical conductivity and high thermal conductivity.

## Methods

### Preparation of Silk Fibroin Solution

Silk Fibroin (SF) solutions were prepared according to reference (*S1*), and the schematic of the preparation is displayed in Figure S1. Bombyx mori cocoons were boiled for 20 min in an aqueous solution of 0.02 M Na_2_CO_3_ and then rinsed thoroughly with deionized water to extract the sericin proteins. After drying, the extracted silk was dissolved in 9.3 M LiBr solution at 60 °C for 4 h, yielding a 20% (w/v) solution. This solution was dialyzed against deionized water using dialysis tube (MWCO 3,500) for 72 h to remove the salt. Then the solution was centrifuged at 9,000 rpm for 20 min at 4 °C to remove silk aggregates formed during the process. The final concentration of silk was about 7 wt%, determined by weighing the remaining solid after drying. The prepared SF solution was stored at 4 °C for future use.

### Preparation of N-graphene sheets/Cu sample

Copper foils (0.25mm-thickness, 99.99%) from Sigma were used as substrate in the experiments. The schematic of the preparation is represented in Figure S2. The surface of copper was treated by hydrogen plasma for 5 minutes under condition of microwave power of 600 W and pressure 10 Torr, and hydrogen flow rate of 30 sccm. Copper foil was coated with the SF solution by spinning at 600 rpm for 2 min, and then put it in a petri dish over 24 hours for natural drying. Before the sample was treated, the surface of sample was covered by a steeliness disk with the size same as the copper substrate. On the opposite edge of the copper substrate, six steeliness screws were used to screwing up. The torsion was measured by a torsionmeter to be 0.2 N·m. And then the dried SF coated copper sample was put in plasma reactor center for following plasma treating. The conditions for treating the sample are: microwave power 800 W, pressure 10 Torr, duration times 10 min, and nitrogen flow rate 50 sccm. No additional heater was employed; the sample was heated by the plasma self-heating. After 10 minutes, the microwave supply was turned off, and the sample was cooled to room temperature under flowing nitrogen with a flow rate of 30 sccm and keeping vacuum pressure at 10–15 torr. The plasma treatment was performed with a home-made reactor with a diameter of 45 mm quartz chamber at 2.45 GHz with microwave generator capable of generating 1.5 kW power which determines the maximum size of the Cu foil that can be used.

### Sample Characterization

The morphologies of nitrogen-doped graphene were examined by a field emission scanning electron microscope (FESEM, JEOL JSM-7800F), and transmission electron microscope (TEM, JEOL JEM2100), respectively. The EELS measurement was performed in the image mode using a Gatan Enfina parallel electron energy loss spectrometer attached to the TEM. The chemical composition was examined by an energy dispersive spectrometer (EDS, Brucker Quantax200). Raman scattering spectra were collected using a Renishaw confocal Raman spectroscope inVia (Renishaw, Gloucestershire GL12 7DW, United Kingdom) with a laser operating at 514.5 nm wavelength and 10 mW power output. Chemical composition and bonding states of samples were characterized using X-ray photoelectron spectroscopy (XPS, Thermo Avantage ESCALAB 250Xi) employing an Al Ka monochromatized radiation as an x-ray source (1,486.6 eV; spot size, 500 μm). The X-ray beam collected C1s, N1s, O1s, and Cu 2p elemental information which rastered over a 500 × 500 μm^2^ areas. For depth profiling measurements, it was accomplished using Ar^+^ ion source and rastered over 2 × 2 mm^2^ area. Sputtering occurred in 15 s intervals. Atomic composition was determined based on photoelectron peak areas and the relative sensitivity factors provide in Thermo Avantage processing software. All data were background subtracted, and charge corrected so that the carbon-carbon bond has a binding energy of 285.0 eV. The completion of etching was defined as the point at which the atomic concentration of C kept stable in the depth-profiling data. The thickness as measured by profilometry was compared with the number of sputter cycles. The speed of the etching was calculated to be 0.19 nm/s.

### Resistivity Measurement

The resistivity of composite samples and copper metal was measured from 100 K to 350 K by a standard dc four-probe method with physical property measurement system (PPMS-9T from Quantum Design, USA). In a typical measurement, a rectangular sample (2 × 6 mm^2^) cut from circular plate was fixed on a sample puck with GE7031 varnish and Kapton film. Four lead wires of Au (Φ 50 μm) were adhered cross the sample with conductive silver paste. Typical exciting current was 4 mA.

### Thermal diffusivity Measurement

The measurements of the thermal diffusivity (***α***) were carried out using “laser flash” method (Netzsch, LFA 467). The “laser flash” technique (LFT) is a transient method that directly measures ***α***. To perform LFT measurement, each sample was positioned on a sample robot, which was surrounded by a furnace. For the measurement, the furnace was held at a predetermined temperature (25–225 °C) and a programmable energy pulse irradiated the back side of the sample, resulting in a homogeneous temperature rise at the sample surface. The resulting temperature rise of the surface of the sample was measured by a very sensitive high speed IR detector. Thus, thermal diffusivity could be determined from the temperature vs. time data (Figure S6). Thermal diffusivity at each temperature was measured for three times, and the ***α*** was obtained by averaging the three values. The deviation of LFT measurement with Netzsch instruments is smaller than 3%. The mass density ***ρ*** of the sample was measured by the Archimedes Method to be about 8.9 g/cm^3^ which is close to the value of oxygen-free copper. The Specific heats (***C***_p_) of the corresponding composite materials were determined in a Shimadzu DSC-50 differential scanning calorimeter (Simadzu Corp., Kyoto, Japan) with computer-aided data analysis, following the procedure described by Casado and Heredia (*S2*). The samples were heated from 25 °C to 225 °C at 5 °C /min. The heat flow into the sample was calculated using the following equation: *d****H****/dt* = ***mC***_p_(*d****T****/dt*) where *d****H****/dt* is the measured heat flow (J/min), ***m*** is the sample mass (g), ***C***_p_ is the specific heat (J/g·K) and *dT/dt* is the scan rate (K/min). Figure S7 presents the result of temperature-dependent ***C***_p_ of N-graphene/Cu composite. Lastly, the thermal conductivity was determined from the equation: ***K* = *ρα C***_**p**_.

## Electronic supplementary material

Supplementary Information

## References

[1] Novoselov KS (2004). Electric Field Effect in Atomically Thin Carbon Films. Science.

[2] Balandin AA (2008). Superior Thermal Conductivity of Single-Layer Graphene. Nano lett..

[3] Hjortstam O, Isberg P, Soderholm S, Dai H (2004). Can we achieve ultra-low resistivity in carbon nanotube-based metal composites?. Appl. Phys. A.

[4] Lee, D. F., Burwell, M. & Stillman, H. Priority research areas to accelerate the development of practical ultraconductive copper conductors. **ORNL/TM-20*****15*****/40**3 (Oak Ridge National Laboratory and the International Copper Association, September 2015).

[5] Subramaniam C (2013). One hundred fold increase in current carrying capacity in a carbon nanotube–copper composite. Nat. Commun..

[6] Baringhaus J (2014). Exceptional ballistic transport in epitaxial graphene nanoribbons. Nature.

[7] Taychatanapat T, Watanabe K, Taniguchi T, Jarillo-Herrero P (2013). Electrically tunable transverse magnetic focusing in graphene. Nat. Phys..

[8] Balandin AA (2011). Thermal properties of graphene and nanostructured carbon materials. Natural materials.

[9] Ramirez S (2017). Thermal and magnetic properties of nanostructured densified ferrimagnetic composites with graphene - graphite fillers. Materials & Design.

[10] Nika DL, Balandin AA (2017). Phonons and thermal transport in graphene and graphene-based materials. Reports on Progress in Physics Physical Society.

[11] Renteria JD, Nika DL, Balandin AA (2014). Graphene thermal properties: Applications in thermal management and energy storage. Applied Sciences.

[12] Seol JH (2010). Two-dimensional phonon transport in supported graphene. Science.

[13] Jagannadham K (2012). Thermal conductivity of copper–graphene composite films synthesized by electrochemical deposition with exfoliated graphene platelets. Metallurgical & Materials Transactions B.

[14] Balandin AA (2009). Chill Out. IEEE Spectrum.

[15] Adamska L, Lin y, Ross AJ, Batzill M, Oleynk II (2012). Atomic and electronic structure of simple metal/graphene and complex metal/graphene/metal interfaces. Phy. Rev. B.

[16] Chen L, Huang Z, Kumar S (2014). Impact of bonding at multi-layer graphene/metal Interfaces on thermal boundary conductance. Rsc Adv..

[17] Goli P (2014). Thermal Properties of Graphene–Copper–Graphene Heterogeneous Films. Nano Lett..

[18] Firkowska I, Boden A, Boerner B, Reich S (2015). The origin of high thermal conductivity and ultra-low thermal expansion in copper-graphite composites. Nano Lett..

[19] Hsieh CC, Liu WR (2017). Synthesis and characterization of nitrogen-doped graphene nanosheets/copper composite film for thermal dissipation. Carbon.

[20] Wu ZS, Ren W, Xu L, Li F, Cheng HM (2011). Doped graphene sheets as anode materials with superhigh rate and large capacity for lithium ion batteries. Acs Nano.

[21] Yu W, Wang L, Qi Y, Wang L, Xie H (2015). The influence of nitrogen doping on thermal conductivity of carbon nanotubes. Thermochimica Acta.

[22] Saito R, Hofmann M, Dresselhaus G, Jorio A, Dresselhaus MS (2008). Raman spectroscopy of graphene and carbon nanotubes. Philos. Trans. R. Sco. A.

[23] Ferrari AC (2006). Raman spectrum of graphene and graphene layers. Phys. Rev. Lett..

[24] Xu E (2010). Doped carbon nanotube array with a gradient of nitrogen concentration. Carbon.

[25] Raul, A. & Loiseau, A. *Heteroatomic Single-Wall Nanotubes Made of Boron*, *Carbon*, *and Nitrogen*. *B-C-N Nanotubes and Related Nanostructures* (Springer New York, 2009).

[26] Hwang JO (2012). N-doped reduced graphene transparent electrodes for high-performance polymer light-emitting diodes. Acs Nano.

[27] Gong C (2010). First-principles study of metal–graphene interfaces. J. Appl. Phys..

[28] Chen L, Huang Z, Kumar S (2013). Phonon transmission and thermal conductance across graphene/Cu interface. App. Phy. Lett..

[29] Boden A, Boerner B, Kusch P, Firkowska I, Reich S (2014). Nanoplatelet size to control the alignment and thermal conductivity in copper-graphite composites. Nano Lett..

[30] Xu Z, Buehler M (2012). Heat dissipation at a graphene-substrate interface. J. Physics-Condensed Matte..

[31] Hwang J (2013). Enhanced Mechanical Properties of Graphene/Copper Nanocomposites Using a Molecular-Level Mixing Process. Adv. Mater..

[32] Shen M, Evans WJ, Cahill D, Keblinski P (2011). Bonding and pressure-tunable interfacial thermal conductance. Phy. Rev..

[33] Chu K (2018). Largely enhanced thermal conductivity of graphene/copper composites with highly aligned graphene network. Carbon.

[34] Costa SD, Weis J, Frank O, Kallac M (2015). Temperature and face dependent copper-graphene interactions. Carbon.

[35] Gong C (2012). Metal-graphene-Metal sandwich contacts for enhanced interface bonding and work function control. ACS Nano..

