# Supplementary material for: N-doped graphene-based copper nanocomposite with ultralow electrical resistivity and high thermal conductivity
Source: Sci Rep. 2018 Jun 18;8:9248. doi: 10.1038/s41598-018-27667-9 (PMC6006153; doi:10.1038/s41598-018-27667-9)
Supplement: Supplementary file 1 — Supplementary Information [file 41598_2018_27667_MOESM1_ESM.pdf]

N-doped graphene-based copper nanocomposite with ultralow electrical resistivity and high thermal conductivity

Liang Zheng[1], Hui Zheng[1], Dexuan Huo[2], Feimei Wu[1], Lihuan Shao[1], Peng Zheng[1], Yuan Jiang[1], Xiaolong Zheng[1], Xinping Qiu[3], Yan Liu[4*], and Yang Zhang[1*]

[1]Laboratory for Nanoelectronics and NanoDevices, School of Electronic Information, Hangzhou Dianzi University, Hangzhou 310018, China.

[2]Institute of Materials Physics, Hangzhou Dianzi University, Hangzhou 310018, China.

[3]Department of Chemistry, Tsinghua University, Beijing 10084, China.

[4]Chemistry and Biochemistry Department, California State Polytechnic University-Pomona, CA 91768, USA.

*Correspondence and requests for materials should be addressed to Y. L and Y. Z (email: yanl@cpp.edu, yzhang09@hdu.edu.cn)

Supplemental Information

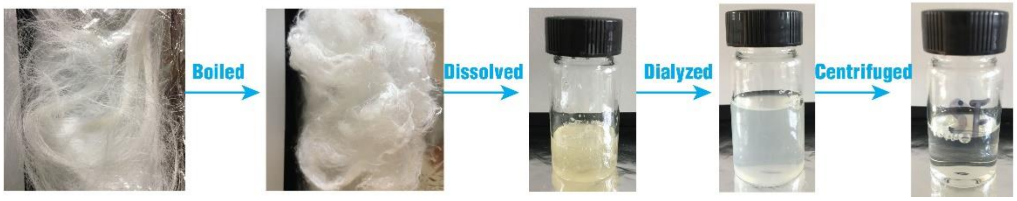

Figure S1. Schematic representation of various steps for silk fibroin solution. (1) Bombyx mori cocoons were boiled for 20 min in an aqueous solution of 0.02 M Na_2CO_3 and then rinsed thoroughly with deionized water to extract the sericin proteins. (2) After drying, the extracted silk was dissolved in 9.3M LiBr solution at 60 ℃ for 4 h, yielding a 20% (w/v) solution. (3) This solution was dialyzed against deionized water using dialysis tube (MWCO 3,500) for 72 h to remove the salt. (4) Then the solution was centrifuged at 9,000 rpm for 20 min at 4 ℃ to remove silk aggregates formed during the process. The final concentration of silk was about 7 wt%, determined by weighing the remaining solid after drying. The prepared SF solution was stored at 4 ℃ for future use.

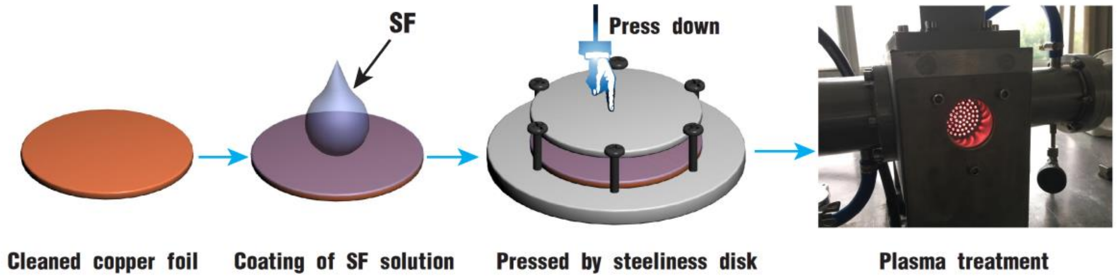

Figure S2. Schematic representation of various steps for NGS-Cu composite fabrication. The surface of copper was treated by hydrogen plasma for 5 minutes under condition of microwave power of 600 W and pressure 10 Torr, hydrogen flow rate of 30 sccm. Copper foil was coated with the SF solution by spinning at 600 rpm for 2min, and then put it in a petri dish over 24 hours for natural drying. Before the sample was treated, the surface of sample was covered by a steeliness disk with the size same as the copper substrate. On the opposite edge of the copper substrate, six steeliness screws were used to screwing up. The torsion is measured by torsionmeter to be 0.2N·m. And then the dried Silk fibroin coated copper sample was put in plasma reactor center for following plasma treating. The conditions for treating the sample are: microwave power 800 W, pressure 10 Torr, duration times 10 min, nitrogen flow rate 50 sccm. No additional heater was employed; sample was heated by the plasma self-heating. After 10 minutes, the microwave supply was turned off, and the sample was cooled to room temperature under flowing nitrogen with a flow rate of 30 sccm and keeping vacuum pressure of 10-15 torr.

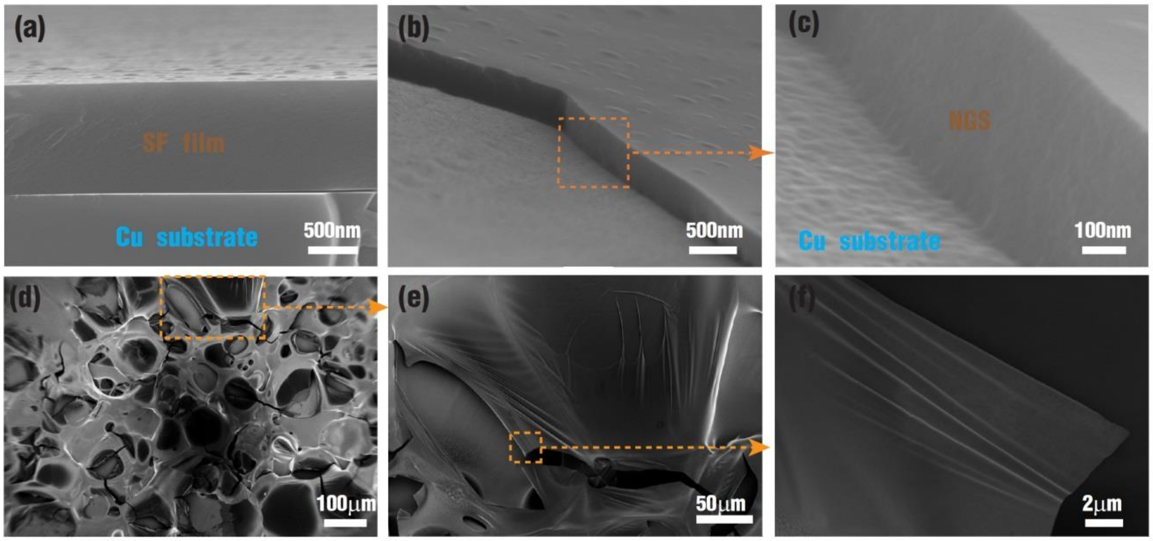

Figure S3. Morphology of the SF-Cu and NGS-Cu composite sample. (**a**) SEM image of the cross section of dried SF-Cu sample. (**b**), (**c**) SEM image of the inclined plane of the NGS-Cu composite sample. The thickness of the dried SF is measured to be ~1.5μm. During plasma treatment, the thickness was gradually reduced due to the vaporing of H_2O. The thickness of NGS is also measured to be ~500nm. Perfect contact between NGS and Cu can be observed in (**c**). (**d**), (**e**) and (**f**) SEM images of the surface of NGS-Cu composite sample. Obviously, the NGS was destroyed by many pores which are attributed to the vaporing of H_2O. In addition, from (**e**), the area of well-shape sheet is larger than 100 μm square which is crucial to apply in electron device. From (**f**), the NGS is a layered structure with crumpled flaky wrinkles.

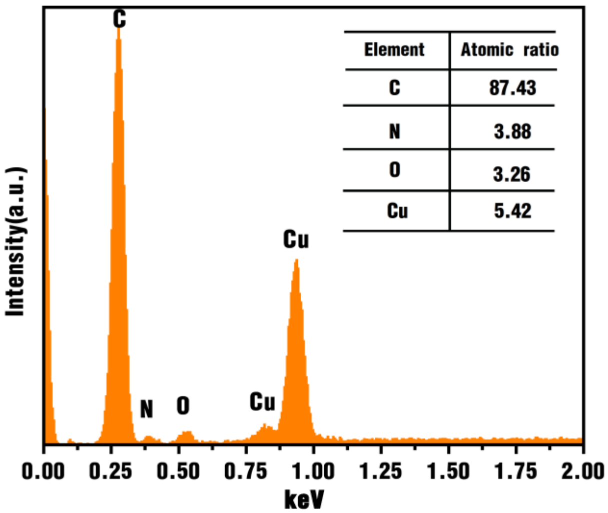

Figure S4. EDS spectrum of the NGS-Cu composite. The spectrum contains strong C and Cu peaks and a small O and N peaks. The oxygen peak is associated with oxygen absorption onto the surface of N-doped graphene. The Cu peak is come from the substrate. The ratio of N/C is 3.88/87.43 (~4.4 $_{atom}$.%) according to the peak area.

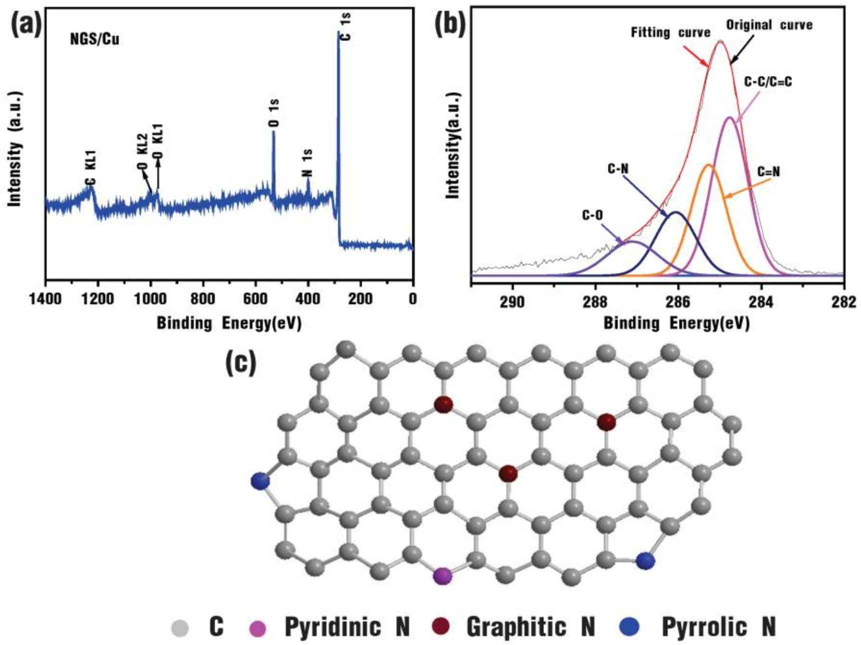

Figure S5. (**a**) XPS full spectra of NGS-Cu composite. A predominant graphite C1s peak at ca. 284eV, N 1s peak at ca. 399 eV and O 1s peak at ca. 531.9 eV are observed. The N doping concentration is 5.2_{atom}.%. (**b**) High-resolution C1s spectra of NGS-Cu composite. It can be divided into four peaks, including C-C/C=C (ca. 284.8eV), C=N (ca. 285.3eV), C=N (ca. 286.1eV), C-O(ca. 287.1eV). The bigger peak C-C/C=C at 284.8eV corresponds to graphite-like sp^2 C, indication most of the C atoms in the NGS are arranged in a conjugated honecomb lattice. The peak C=N at 285.8eV is identified as C-N bonding structures, with sp^2 hybridized carbon. The existence of bonding between C and O atoms is attributed to the oxidation on edge and defect positions or the O absorption onto the surface of NGS. (**c**) The schematic representation of NGS. The grey, pink, red and blue spheres circled by dotted line represent the C, pyridinic N, quaternary N and pyrrolic N atoms, respectively.

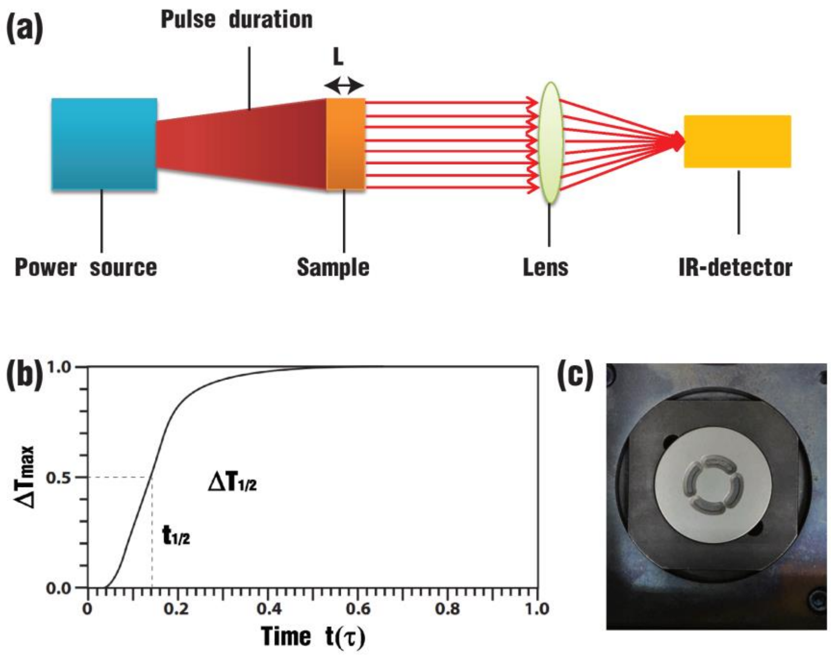

Figure S6. Schematic of thermal diffusivity measurement and picture of testing apparatus. (**a**) Schematic of thermal diffusivity measurement. A flash of a xenon lamp (wavelength λ=150-2000nm) with the laser voltage of 230V for 0.02ms is using Power Source and Pulse duration. The measured sample is NGS-Cu composite with 0.25mm-thickness (L) and 25.4mm-diameter. Here, the thickness of NGS (~500nm) can be neglected. (**b**) Theoretically curve of Temperature vs. Time (T~t). The thermal diffusivity can be calculated as: (1) Determine the baseline and maximum rise to give the temperature difference, T_{max}; (2) Determine the time required from the initiation of the pulse for the rear force temperature to reach $T_{1/2}$. This is the half time, $t_{1/2}$. (3) Calculate the thermal diffusivity, α, from the samples hight L and the halftime $t_{1/2}$, as follows: α=0.13879L^2/ $t_{1/2}$. (**c**) The picture of testing apparatus.

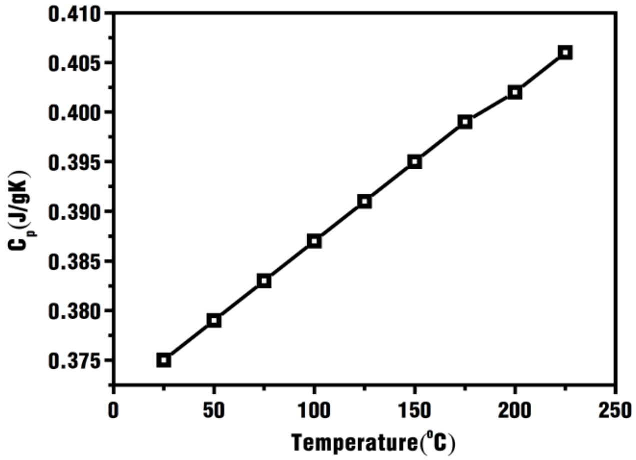

Figure S7. The temperature-dependent $\boldsymbol{C}_\mathrm{p}$ of NGS-Cu composite.

References

S1. D. N. Rockwood, R. C. Preda, T. Yucel, X. Q. Wang, M. L. Lovett, D. L. Kaplan, *Nat. Protoc.* **10**, 1612-1631(2011).

S2. C. G. Casado, A. Heredia, *BBAs Biomembranes* **1511**, 291-296 (2001).
